# Influenza Virus Infection in Racing Greyhounds

**DOI:** 10.3201/eid1112.050810

**Published:** 2005-12

**Authors:** Kyoung-Jin Yoon, Vickie L. Cooper, Kent J. Schwartz, Karen M. Harmon, Won-II Kim, Bruce H. Janke, Jody Strohbehn, Darcey Butts, Joe Troutman

**Affiliations:** * Iowa State University, Ames, Iowa, USA; † Strohbehn Veterinary Clinic, Council Bluffs, Iowa, USA; ‡ Glenwood Veterinary Clinic, Glenwood, Iowa, USA; § Dubuque Greyhound Track, Dubuque, Iowa, USA

**Keywords:** hemorrhagic pneumonia, influenza virus, canine, H3N8 subtype, greyhounds, letter

**To the Editor:** Influenza is globally the most economically important respiratory disease in humans, pigs, horses, and fowl (*1*). Influenza virus is known for its continuous genetic and antigenic changes, which impedes effective influenza control (*1**,**2*). More importantly, emergence of a new subtype by genetic reassortment or interspecies transmission is of great concern for preventing influenza epidemics and pandemics (*1*). Recently, influenza outbreaks have occurred in species (feline and canine) that historically do not carry influenza virus (*3**,**4*), which alerted both regulatory and scientific communities to expansion of the host range of influenza virus. We report an outbreak of respiratory disease by influenza virus infection in Iowa racing greyhounds after influenza outbreaks in Florida in 2004.

In mid-April, an influx of racing greyhounds into Iowa greyhound tracks resulted in outbreaks of respiratory disease within the track compounds. The disease was characterized by rapid onset of fever and cough, rapid respiration, and hemorrhagic nasal discharge. The illness rate was almost 100% in both racetrack compounds, although the death rate was <5%. Most affected dogs recovered, yet many died of hemorrhagic pneumonia. Therapeutic administration of broad-spectrum antimicrobial drugs reduced the severity of the disease but could not control it.

Tissue samples from 4 animals that died of severe pneumonia were submitted to the Iowa State University Veterinary Diagnostic Laboratory. The animals represented 2 different racing tracks located in eastern and western Iowa. On gross examination, lungs exhibited extensive red to red-black discoloration with moderate to marked palpable firmness. Mild fibrinous pleuritis was also noted. Microscopically, lung sections were characterized by severe hemorrhagic interstitial to bronchointerstitial pneumonia. Patchy interstitial change with alveolar septal thickening, coagula of debris in alveoli, and associated atelectasis were evident. Focally extensive pyogranulomatous bronchointerstitial pneumonia with dilatation of airways by degenerate cells and debris was observed. Scattered vasculitis and vascular thrombi were apparent.

Microbiologic testing for conventional viral and bacterial agents did not show any important pathogens except *Streptococcus equi* subsp. *zooepidemicus* from lung tissues of all animals examined. Two of the 4 lung samples were positive for influenza A virus by a real-time reverse transcription–polymerase chain reaction (RT-PCR) (*5*). Viral pneumonic lesions of both lungs were positive for immunohistochemistry (IHC) with monoclonal antibody specific for the nucleoprotein of influenza A virus (*6*) and with antigen-capturing enzyme-linked immunosorbent assay (*Direct*igen Flu A, Becton-Dickinson, Sparks, MD, USA). Bronchioalveolar lavage samples from the 2 positive lungs were also positive by RT-PCR for influenza A virus.

Virus isolation was attempted; the influenza virus in canine lungs was unexpected since no influenza virus infection in dogs had been reported, except a recent communication at a meeting of veterinary diagnosticians (*4*). A virus that was able to agglutinate rooster erythrocytes was isolated in Madin-Darby canine kidney cells from lung and bronchioalveolar lavage fluid of 1 of the 2 animals in which influenza virus was detected by IHC and RT-PCR. Isolates were determined by RT-PCR to be influenza A virus of H3 subtype. The US Department of Agriculture National Veterinary Services Laboratory (Ames, IA) subtyped the virus isolates (A/Canine/Iowa/13628/2005) as H3N8 by using hemagglutination-inhibition assay and neuraminidase-inhibition assay.

Sequencing hemagglutinin (HA) and neuraminidase (NA) genes of both isolates showed 100% and 99.8% identity, respectively, between the 2 isolates. Phylogenetically, the HA gene (GenBank accession no. DQ146419) of the isolates was genetically close (96%–98% nucleotide homology) to the HA gene of recent H3N8 equine influenza viruses (*7*). The NA gene (DQ146420) of the isolates also showed 96%–98% homology with the NA gene of recent H3N8 equine influenza viruses. Internal genes remain to be sequenced.

In conclusion, recent outbreaks of hemorrhagic pneumonia and associated deaths in Iowa racing greyhounds were primarily due to infection by an H3N8 influenza virus genetically and antigenically similar to equine influenza viruses. This conclusion can be supported by a previous report of fatal hemorrhagic pneumonia by H3N8 virus infection in racing greyhounds in Florida (*4*). The fact that greyhounds in 2 different racetracks, which are in geographically remote sites in Iowa, simultaneously died of the disease without the involvement of sick horses suggests that the influenza virus isolate is likely a canine-adapted strain and able to perpetuate and spread among dogs. While influenza virus infection was likely responsible for the disease outbreaks, the contribution that *S. zooepidemicus* might have made to the disease and the severity of clinical manifestations remains to be further evaluated since the bacterium has been implicated in respiratory disease and septicemia-associated problems in many different animal species (*8**,**9*).
